# Supplementary material for: Genome-wide association across Saccharomyces cerevisiae strains reveals substantial variation in underlying gene requirements for toxin tolerance
Source: PLoS Genet. 2018 Feb 23;14(2):e1007217. doi: 10.1371/journal.pgen.1007217 (PMC5849340; doi:10.1371/journal.pgen.1007217)
Supplement: S2 Table — SNP classifications were performed by SnpEff as outlined in Methods. Low impact genic polymorphisms are represented by synonymous codon changes, moderate impact genic SNPs are nonsynonymous codon changes, and high impact variants include introduction of premature stop codons, altered start position, or interruptions of slicing regions. (DOCX) [file pgen.1007217.s011.docx]

| **Type** | **Number of SNPs** |
| --- | --- |
| Low | 179,662 |
| Intergenic | 179,195 |
| Moderate | 126,030 |
| High | 1,415 |

**S2 Table. Summary of SNPs and predicted impacts.** SNP classifications were performed by SnpEff as outlined in Methods. Low impact genic polymorphisms are represented by synonymous codon changes, moderate impact genic SNPs are nonsynonymous codon changes, and high impact variants include introduction of premature stop codons, altered start position, or interruptions of slicing regions.
