# Supplementary material for: Genome-wide association across Saccharomyces cerevisiae strains reveals substantial variation in underlying gene requirements for toxin tolerance
Source: PLoS Genet. 2018 Feb 23;14(2):e1007217. doi: 10.1371/journal.pgen.1007217 (PMC5849340; doi:10.1371/journal.pgen.1007217)
Supplement: S5 Table — Strain genotype sources for cloning of tolerant and sensitive allele for plasmid complementation are shown. (DOCX) [file pgen.1007217.s014.docx]

**S5 Table. Plasmids with tolerant and sensitive alleles.** Strains used to amplify the tolerant and sensitive allele for plasmid complementation are shown.

| **Gene** | **Tolerant Allele** | **Sensitive Allele** |
| --- | --- | --- |
| LEU3 | YPS128 | 378604X |
| SAP190 | YPS128 | YJM1419 |
| DAT1 | YPS128 | YJM1444 |
| TIR3 | YPS128 | YJM1444 |
| RPL21 | YPS128 | YJM1444 |
| FLO10 | YPS128 | 378604X |
| SHE1 | YPS128 | 378604X |
| FLO1 | YPS128 | YJM1444 |
