# Supplementary material for: Genome-wide association across Saccharomyces cerevisiae strains reveals substantial variation in underlying gene requirements for toxin tolerance
Source: PLoS Genet. 2018 Feb 23;14(2):e1007217. doi: 10.1371/journal.pgen.1007217 (PMC5849340; doi:10.1371/journal.pgen.1007217)
Supplement: S6 Table — The type of allele that is tested in each hybrid is labeled as sensitive (S) and tolerant (T). (DOCX) [file pgen.1007217.s015.docx]

**S6 Table. Strains used for RHA between YPS128 and YJM1444 to test alleles found in GWAS.** The type of allele that is tested in each hybrid is labeled as sensitive (S) and tolerant (T).

| **RHA** | **Parent #1; MAT a** | **Parent #2; MAT α** |
| --- | --- | --- |
| *ALD3^S^* | YPS128 (HO::*KAN-MX*; ald3::*HERP1.1*) | YJM1444 (HO::*NAT-MX*) |
| *DAT1^S^* | YPS128 (HO::*KAN-MX*; dat1:*HERP1.1*) | YJM1444 (HO::*NAT-MX*) |
| *PIG1^S^* | YPS128 (HO::*KAN-MX*; pig1:*HERP1.1*) | YJM1444 (HO::*NAT-MX*) |
| *MNE1^S^* | YPS128 (HO::*KAN-MX*; mne1:*HERP1.1*) | YJM1444 (HO::*NAT-MX*) |
| *RPL21B^S^* | YPS128 (HO::*KAN-MX*; rpl21b:*HERP1.1*) | YJM1444 (HO::*NAT-MX*) |
| *MNE1^T^* | YJM1444 (HO::KAN-MX; mne1:*HERP1.1*) | YPS128 (HO::*NAT-MX*) |
| *FLO1^T^* | YJM1444 (HO::KAN-MX; flo1:*HERP1.1*) | YPS128 (HO::*NAT-MX*) |
| *FLO1^S^* | YPS128 (HO::*KAN-MX*; flo1:*HERP1.1*) | YJM1444 (HO::*NAT-MX*) |
| *KDX1^T^* | YJM1444 (HO::KAN-MX; kdx1:*HERP1.1*) | YPS128 (HO::*NAT-MX*) |
| *UBP5^T^* | YJM1444 (HO::KAN-MX; ubp5:*HERP1.1*) | YPS128 (HO::*NAT-MX*) |
| *RIM20^T^* | YJM1444 (HO::KAN-MX; rim20:*HERP1.1*) | YPS128 (HO::*NAT-MX*) |
| *ALD3^T^* | YJM1444 (HO::KAN-MX; ald3:HERP1.1) | YPS128 (HO::*NAT-MX*) |
| *UBP5^S^* | YPS128 (HO::*KAN-MX*; ubp5:*HERP1.1*) | YJM1444 (HO::*NAT-MX*) |
| *KDX1^S^* | YPS128 (HO::*KAN-MX*; kdx1:*HERP1.1*) | YJM1444 (HO::*NAT-MX*) |
| *TIR3^S^* | YPS128 (HO::*KAN-MX*; tir3:*HERP1.1*) | YJM1444 (HO::*NAT-MX*) |
| *RIM20^S^* | YPS128 (HO::*KAN-MX*; rim20:*HERP1.1*) | YJM1444 (HO::*NAT-MX*) |
